# Predictors of expectant fathers’ parental leave-taking intentions before birth: masculinity, fatherhood beliefs, and social support

**DOI:** 10.3389/fpsyg.2024.1247193

**Published:** 2024-02-12

**Authors:** Carolin Scheifele, Colette Van Laar, Melanie C. Steffens

**Affiliations:** ^1^Center for Social and Cultural Psychology, University of Leuven, Leuven, Belgium; ^2^PhD Fellow of the Research Foundation–Flanders, Brussels, Belgium; ^3^Department of Social, Environmental, and Economic Psychology, RPTU Kaiserslautern-Landau, Landau, Germany

**Keywords:** parental leave, transition to parenthood, masculinity, fatherhood, social support

## Abstract

Despite continuing progress, men remain underrepresented in childcare, domestic labor, and other care work. Because parental leave is discussed as a gateway to increasing men’s childcare engagement, we aimed to gain insights into predictors of men’s parental leave-taking intentions during the transition to parenthood. Using outcomes on a continuum from behavioral preferences to more behavior-oriented measures, we examine how masculinity and fatherhood beliefs as well as social support become relevant during men’s formation of their leave-taking intentions. Planned analyses of data collected from 143 expectant fathers in Belgium and Germany revealed that the support men perceive from their partners for taking leave predicts their parental leave-taking desire, intention, and planned length of leave. Moreover, men’s conception of a prototypical man, especially in terms of agency, was linked to their desire to take leave. Against expectations, father role attitudes and workplace support did not emerge as relevant predictors of men’s intended leave-taking. Results of exploratory analyses suggest that care engagement of peers, expected backlash, and self-efficacy beliefs additionally play a role in men’s intended leave-taking. We discuss parental leave as a negotiation process within couples and review the role of men’s normative environment for their intended leave-taking.

## 1 Introduction

Involved, caring, and new – these are some of the terms that are frequently used when talking about fatherhood today. In fact, the shift towards a fatherhood ideal that expects fathers to be more involved in childcare and to develop closer emotional bonds with their children is not exactly new anymore but was already observed in Western cultures since the 1980s (Wall and Arnold, 2007; Dermott and Miller, 2015). Indeed, fathers have increased their engagement in childcare and household labor and continue to do so (Altintas and Sullivan, 2016, 2017). For example, more and more fathers across Europe are making use of their parental leave entitlement (Eurofound, 2019), and roughly a third of fathers in Belgium and Germany takes parental leave (Samtleben et al., 2019b; Koslowski et al., 2022). Nevertheless, women continue to be more affected by the transition to parenthood and after becoming a parent often reduce their work hours while increasing time spent on childcare and household tasks (Abele and Spurk, 2011; Baxter et al., 2015). Women across cultural contexts also at a young age already have higher intentions than men to take parental leave (Olsson et al., 2023) and continue to be overrepresented relative to men in actual leave uptake (Koslowski et al., 2022). A more equal share of parental leave among women and men has been discussed as a way to promote gender equality (Castro-García and Pazos-Moran, 2016; Meeussen et al., 2020), especially during the transition to parenthood when gender-role attitudes and the gendered division of labor tend to become more traditional (Baxter et al., 2015). In addition, men’s increased care engagement can have benefits on various levels, for example, for their own well-being, their partners’ career advancement, and their children’s developmental outcomes (for an overview, see Croft et al., 2015; Meeussen et al., 2020). Men’s parental leave-taking specifically can lead to fathers being more involved in childcare later on (Meil, 2013; Almqvist and Duvander, 2014; Bünning, 2015; Petts and Knoester, 2018).

Various reasons for men’s comparatively low interest in and uptake of parental leave have been discussed in the literature. Whereas external barriers such as the lack of sufficient income replacement during leave are often emphasized (e.g., Castro-García and Pazos-Moran, 2016; Karu and Tremblay, 2018; Kaufman, 2018), a recent examination of young men’s (and women’s) intentions to take parental leave across 37 nations suggests that individual-level factors such as men’s gender role attitudes outweigh country-level factors such as specific leave policies (Olsson et al., 2023). The goal of the current study is to have a closer look at such psychological contributors to men’s parental leave-taking intentions before birth. By examining leave-taking *intentions*, we learn more about precursors of men’s leave-taking and possible pathways for interventions. Moreover, we examine the different layers of men’s intended leave-taking, namely whether they desire to take leave, whether they intend and plan to do so, and if so, for how long. We assume that these dependent variables form a continuum from behavioral preferences to behavioral intentions (Bagozzi, 1992; Perugini and Bagozzi, 2001) and thus provide more insights into predictors of men’s intended leave-taking at various stages in their decision-making process. In addition, examining the hypothesized relations cross-sectionally will provide suggestive evidence as to whether the relations can also be expected longitudinally. Furthermore, we contribute to the current literature by simultaneously considering men’s gender beliefs regarding what constitutes a prototypical, ideal man and gender *role* beliefs regarding men’s role as a father for their intended leave-taking. Accounting for the normative environment men find themselves in, we additionally focus on how active support or discouragement from relevant others is related to men’s intended leave-taking.

A starting point for understanding men’s interest in care roles generally and parental leave specifically are gender norms and stereotypes (see Croft et al., 2015; Meeussen et al., 2020). According to social role theory (Eagly, 1987; Eagly and Wood, 2012), such gendered beliefs develop from observing a gendered division of labor and deriving expectations about male and female traits and behaviors. Gender stereotypes can be divided into two fundamental content dimensions: agency and communion (Bakan, 1966; Abele and Wojciszke, 2014). Traditionally, gender stereotypes ascribe agentic traits and behaviors to men (e.g., being independent, assertive, or competent) and communal traits and behaviors to women (e.g., being warm, caring, or helpful; Bakan, 1966; Burgess and Borgida, 1999; Prentice and Carranza, 2002). However, recent examinations of change in gender stereotypes found that men’s self-descriptions are becoming less stereotypic and that men do associate themselves with communion (Hentschel et al., 2019). Other findings suggest that women and men do not ascribe communion more to men now than in the past and that women’s higher scores on communion persist or have even increased (Hentschel et al., 2019; Eagly et al., 2020). Given the ambiguity in change of gender stereotypes, an important source of men’s interest in communal, care-oriented engagement is what *they* perceive as desirable and normative for their gender group. We, therefore, examine men’s conception of a prototypical man, the ideal-type member of their gender group (Oakes et al., 1998; Wenzel et al., 2007). Prototypes, as described in self-categorization theory (Turner et al., 1987), have conceptual similarity to constructs such as stereotypes or norms but better capture an *individual’s* perception of a prototypical member of their gender group (see Hogg et al., 2012). Such notions of what it means to be a man have already been examined from a sociological and qualitative perspective with regard to men’s parental leave-taking (Brandth and Kvande, 1998; Almqvist, 2008; Johansson, 2011; Schmidt et al., 2015). For example, in a study conducted in Austria, fathers’ parental leave-taking decisions were made within work-focused masculinity ideals and depended on fathers’ personal wishes and whether external circumstances allowed for leave (Schmidt et al., 2015). Moreover, Norwegian fathers who felt like they did not have to prove their masculinity were more content during leave but also kept strong ties to their breadwinning role (Brandth and Kvande, 1998). Thus, first evidence of how masculinity is constructed in relation to men’s parental leave-taking exists, but we know less about how male gender stereotypes and gender norms contribute to whether men intend to take leave. From research on father involvement more generally, we know that less traditional masculinity norms are related to more care-oriented father involvement, such as showing more warmth and using less harsh discipline (Petts et al., 2018; Shafer et al., 2020). In the present research, we aim to shed light on whether less traditional (i.e., more communal and less agentic) notions of masculinity are also related to an important precursor of father involvement, namely men’s intended leave-taking. Thus, we examine the link between intended leave-taking and the degree to which men associate a prototypical man with the stereotypic dimensions of agency and communion (Bakan, 1966; Abele and Wojciszke, 2014).

When men become fathers, they not only face masculinity ideals but also ideals regarding fatherhood. In fact, the father role could provide leeway for men to engage in caretaking as stereotypes of fathers are less restrictive in terms of communal aspects than those of men (Park and Banchefsky, 2018; Ciaccio et al., 2021). These differing perceptions of men and fathers are likely based on the added social role of being a parent, a role that implies some degree of communion and caretaking. Thus, in addition to examining men’s conception of their gender group and which attributes constitute a prototypical man, we examine men’s gender *role* of being a father and their attitudes towards this role. First evidence for the relevance of gender role attitudes for men’s leave-taking exists across national contexts such as Sweden, the United States, and Germany. Generally, less traditional gender role attitudes were related to higher intentions to take leave, higher chances to do so, and longer leave length (Hyde et al., 1993; Vogt and Pull, 2010; Duvander, 2014; Olsson et al., 2023). However, in more recent research men’s leave length was neither predicted by their own nor by their partners’ gender role attitudes (in a United States context and German-speaking countries; Stertz et al., 2017; Berrigan et al., 2021). An explanation could be the ambiguous measurement of gender role attitudes in some of these studies, which mostly included attitudes towards women’s gender roles (Hyde et al., 1993; Stertz et al., 2017; for an exception, see Vogt and Pull, 2010). Yet, how men see their own role as a father could be more closely related to their parental leave-taking intentions. In addition, fatherhood does not have to be defined on a continuum from breadwinning to caregiving, but men could see their responsibility in and identify with both. Thus, in the current study we examine father role attitudes towards breadwinning and childcare separately (as suggested by Hyde et al., 1993).

Men’s parental leave-taking decision is, furthermore, shaped within a normative environment in which social support (or lack thereof) can signal whether others approve or disapprove of their communal engagement. As communal engagement is traditionally counter-stereotypic for men, men can fear backlash and negative consequences, such as experiencing stigma or career disadvantages for wanting to take leave (see role congruity theory, Eagly and Karau, 2002; Rudman and Mescher, 2013; Miyajima and Yamaguchi, 2017). However, when others signal that they support men’s leave-taking, this challenges what is perceived as normative and can alleviate such threat (for first evidence on social support and men’s communal orientation, see Schreiber et al., 2023).

For parental leave-taking decisions, especially the interactions and support between partners plays a crucial role. In fact, negotiations are often focused on the partner’s wishes (McKay and Doucet, 2010; Beglaubter, 2017; Kaufman and Almqvist, 2017; for an exception, see Schmidt et al., 2015), especially when there is no earmarked leave available for fathers (McKay and Doucet, 2010; Castro-García and Pazos-Moran, 2016). Nevertheless, mothers have been found to encourage fathers to take longer leaves to achieve a more equal division of childcare and foster the bonding between father and child (Kaufman and Almqvist, 2017). More generally, when mothers encouraged childcare efforts, fathers’ relative involvement as reported by both parents was higher, and fathers perceived that they had a greater say in decisions regarding the child’s health (Schoppe-Sullivan et al., 2008; Zvara et al., 2013). Besides their partners and others around them, men’s normative environment and leave-taking decisions are additionally shaped by their workplace. As a general trend, organizations are becoming more supportive of men’s leave-taking (Haas and Hwang, 2009; Brandth and Kvande, 2019). Moreover, colleagues can be a facilitator of men’s leave-taking as men are more likely to take longer leave if colleagues have done so before them (Bygren and Duvander, 2006). However, in organizations that emphasize ideal worker norms (i.e., prioritizing work over family and aiming for high workload and output), men are less likely to take (longer) leave and report more negative career consequences if they still do so (Haas et al., 2002; Haas and Hwang, 2019; Samtleben et al., 2019a).

Taken together, we investigate predictors of men’s intended parental leave-taking before birth, with a focus on men’s conception of a prototypical man, father role attitudes, and social support. As outcomes, we look at expecting fathers’ general intentions to take leave, their desire to do so, as well as for how long they expect to take leave (summarized as *intended parental leave-taking* in the following). Looking at men’s conception of a prototypical man, we expect communal prototypes of men to be positively related to men’s intended parental leave-taking (H1.1), whereas agentic prototypes of men should be negatively related to men’s intended parental leave-taking (H1.2). Likewise, we expect father role attitudes regarding childcare to be positively related to men’s intended parental leave-taking (H2.1), whereas father role attitudes regarding breadwinning should be negatively related to men’s intended parental leave-taking (H2.2). Lastly, we investigate the role of men’s personal environment in their intended leave-taking. We expect partner support (H3.1) and workplace support (H3.2) for leave-taking to be positively related to men’s intended parental leave-taking.

## 2 Materials and methods

The study was preregistered on Aspredicted^1^ and received ethical approval from the Social and Societal Ethics Committee of the University of Leuven. We describe deviations from the preregistration and further included measures in Supplementary material.

### 2.1 Procedure and context of data collection

We collected data from men in Belgium and Germany who were expecting their first child. Participants were asked to complete an online survey around 3 months before birth.^2^ Importantly, different national policies for protected paid leave apply in Belgium and Germany. In Belgium, men can take parental leave (“*ouderschapsverlof”*) for 4 months, and this leave cannot be transferred between partners. Part-time leave regulations are available, but income replacement (provided through government funding) is comparatively low, with roughly 800€ per month for full-time leave (Koslowski et al., 2022; RVA, 2022).^3^ In 2021, 34% of leave-takers in Belgium were fathers (vs. mothers) who predominantly used it as a flexibility measure to combine work and family. Sixty-three percent of fathers took 1 day of leave per week, and 20% took half a day per week or 1 day every 2 weeks (Koslowski et al., 2022). In Germany, parents can divide paid parental leave (“*Elterngeld*”) of up to 12 months between each other, with an additional period of 2 months not transferrable to the other parent. Regulations for part-time leave also exist, and combining work and childcare is encouraged by an additional 4 months of part-time leave if both parents work part-time. Income replacement is higher than in Belgium, with parents receiving 65% of the average Net income of the last 12 months before the birth (capped at 1800€, provided through government funding; BMFSFJ, 2022; Koslowski et al., 2022). In 2016, 37% of fathers took parental leave in Germany. However, in 2018, 72% of those took parental leave at most for the duration of the non-transferable period of 2 months (Samtleben et al., 2019b).

We recruited participants through people and places that we expected to be in touch with expectant parents (e.g., prenatal classes, hospitals, gynecology practices, midwives, shops for baby equipment, parenting and baby fairs, professional organizations for midwives or gynecologists, companies in male-dominated industries etc.). Furthermore, we used social media (Facebook, Instagram, and Twitter) and encouraged snowball sampling. We invited participants to take part in a study on how the birth of the first child affects the work and family situation of men (and their partners). At the beginning of the online survey, participants received a detailed information letter on the procedure of the study and gave informed consent online. Afterwards, we assessed and implemented the exclusion criteria specified above. Eligible participants then read a short summary of the current leave policies in their respective countries before completing the main survey measures, suspicion and quality checks, and demographic information. At the end, participants could indicate special circumstances of, for example, their work or family situation. Lastly, we thanked participants and asked them for help with recruiting additional participants. For each referred participant who filled in the first survey, participants (and others) could receive a 10€ gift card. Moreover, participants themselves received a 10€ gift card for each completed survey and had the chance to win a family weekend trip at the end of the study.

### 2.2 Sample and sensitivity analysis

In total, 171 participants completed the survey who met the preregistered criteria of identifying as male, being at least 18 years old, expecting their first child, and being eligible to receive parental or paternity leave. We excluded the data of eight participants from the analyses because they failed attention or quality checks. We also excluded 20 multivariate outliers based on the MCD75 (Minimum Covariance Determinant with a breakdown point of 0.25), with a chi-square at *p* = 0.001 (Leys et al., 2019; see Supplementary material for results including outliers). Among the final 143 participants, 115 resided in Belgium and 28 in Germany. Participants were, on average, 31 years old (*SD* = 3.60; range: 25–42). Most were married (69%) or in a committed relationship (26%) and identified as heterosexual (98%; 2% identifying as bisexual). Participants were, on average, highly educated, with 43% having a university degree, 27% higher professional education, and 17% secondary education. In terms of relative income, 18% had a much higher income than their partner, 35% a higher income, 23% more or less equal income, and 15% a lower income than their partner. They worked, on average, 41 h per week (*SD* = 7.32), and the majority did not have any leadership responsibility (66%). Their political orientation was moderate to slightly left (*M* = 4.56 on a 9-point scale, *SD* = 1.65), and they were not religious on average (*M* = 2.48 on a 9-point scale, *SD* = 2.07).

We conducted a sensitivity analysis with G*Power 3 (Faul et al., 2007) to learn which effect sizes we were able to detect given a sample size of *N* = 143 (*α* = 0.05, 1 - *β* = 0.95). In analyses with up to 11 predictors, we were able to detect effect sizes for regression coefficients of *f*^2^ = 0.09 (i.e., small- to medium-sized effects).

### 2.3 Measures

Unless otherwise indicated, we used 7-point scales ranging from 1 = “strongly disagree” to 7 = “strongly agree.” For measures we suspected to be prone to ceiling effects (and, for consistency, for those situated in close proximity to them within the survey), we implemented 9-point scales to ensure adequate differentiation at the higher end of the scale.

#### 2.3.1 Prototypes of men

We assessed participants’ idea of a prototypical man by asking what it means to them to be a man and to what extent four agentic (e.g., assertive, *α* = 0.64) and six communal (e.g., compassionate, *α* = 0.77) traits describe an ideal man in their opinion [adapted from Van Grootel et al. (2018) and Hentschel et al. (2019); see Supplementary material for results excluding items for which no gender differences were found in past research]. We used a 7-point scale from 1 = “not at all” to 7 = “very much.”

#### 2.3.2 Father role attitudes

We asked participants what it means to them to be a father and how they see the responsibility of a father for his child, adapted from the Caregiving and Breadwinning Identity and Reflected-Appraisal Inventory (CBIRAI; Maurer et al., 2001; using a 9-point scale from 1 = “strongly disagree” to 9 = “strongly agree”). Five items focused on physical and social caregiving, with only two items sufficiently correlated to form a scale (*r* = 0.66; e.g., “A father should NOT be very involved in the day-to-day matters of caring for his child.”; recoded). Four items formed a scale focusing on breadwinning (*α* = 0.65; e.g., “A father has a strong responsibility as a parent to be the financial provider for his family.”). The results of factor analyses can be found in Supplementary material.

#### 2.3.3 Social support for leave-taking

We measured the social support men perceived with one item pertaining to the support from their partner and one from people at work (e.g., their boss or colleagues). Participants indicated how much support or discouragement they experienced from their partner [people at work] to take up parental leave adapted from Schreiber et al. (2023) on a 9-point scale (1 = “lots of discouragement,” 5 = “neither much discouragement nor support,” 9 = “lots of support”).

#### 2.3.4 Others’ leave-taking, others’ childcare engagement, expected backlash for leave-taking, expected parental self-efficacy

We included additional predictors in the analyses that have been linked to men’s parental leave-taking before. Focusing on men’s personal environment, we asked participants how many men in their surroundings who became fathers during the past years took parental leave (9-point scale from 1 = “very few” to 9 = “almost all”) and how much these fathers engage in childcare (9-point scale, 1 = “very little as compared to their partner,” 5 = “as much as their partner,” 9 = “much more than their partner”). For expected backlash effects, participants answered the item “I worry about being labeled negatively for putting my career on hold to care for my young child.” Adapted from Rudman and Fairchild (2004) and Vogt and Pull (2010), omitting a second item due to low correlation (for links to men’s leave-taking, see Samtleben et al., 2019a). Lastly, we measured expected self-efficacy for childcare with two items [*r* = 0.82; e.g., “I feel like I will be capable of taking care of my child.”; adapted from Črnčec et al. (2008)]. Although general self-efficacy beliefs were not related to men’s leave-taking (Horvath et al., 2018), evidence exists for the relation between *parental* self-efficacy and father involvement as well as parental competence (Jones and Prinz, 2005; Trahan, 2018).

#### 2.3.5 Intended parental leave-taking

We measured men’s intended leave-taking via three operationalizations: desired parental leave-taking, parental leave-taking intentions, and expected length of parental leave. We assessed desired parental leave-taking with one item (“I would like to take leave.”), adding two items on parental leave-taking intentions [*r* = 0.88; e.g., “I intend to take leave.”; adapted from Yzer (2012) and Miyajima and Yamaguchi (2017)]. For the expected length of parental leave, participants indicated how long they expected to take parental leave in full-time weeks (Belgium) or months (Germany). Those planning to take leave part-time thus recalculated their intended length into full-time weeks or months. We then calculated a percentage measure, indicating how much of the available leave participants expected to take (see Supplementary material for results using absolute expected leave lengths).

## 3 Results

### 3.1 Descriptive statistics

Table 1 shows means, standard deviations, and correlations for all predictors and dependent variables. Notable here are the high means for father role attitudes regarding childcare and support from the partner for taking leave, suggesting a comparatively egalitarian sample. Moreover, participants had a relatively strong wish to take parental leave, whereas average leave-taking intentions were slightly lower. On average, participants expected to take roughly 58% of the available leave length. Descriptive statistics per country of data collection can be found in Supplementary Table S1.

**Table 1:** Means, standard deviations, and correlations of study variables.

	*M* (*SD*)	Correlations (*N* = 124–143)											
		2.	3.	4.	5.	6.	7.	8.	9.	10.	11.	12.	13.
Prototypes of men—communion^a^	5.10 (0.79)	0.22**	0.13	0.07	0.10	0.17*	0.11	0.07	−0.09	0.20*	0.26**	0.15^†^	0.10
Prototypes of men—agency^a^	5.21 (0.82)		−0.03	0.22**	0.11	0.05	−0.05	0.05	0.10	0.07	−0.04	−0.03	−0.16^†^
Father role attitudes— childcare^b^	8.22 (0.95)			−0.10	0.29***	0.08	−0.03	−0.05	−0.09	0.13	0.15^†^	0.15^†^	0.08
Father role attitudes— breadwinning^b^	4.46 (1.53)				−0.31***	−0.01	−0.19*	0.23**	0.05	−0.17*	−0.15^†^	−0.22*	−0.27**
Partner support^b^	7.89 (1.50)					0.35***	0.23**	−0.10	−0.08	0.17^†^	0.48***	0.45***	0.25**
Workplace support^b^	6.36 (1.76)						0.36***	−0.04	−0.37***	0.12	0.24**	0.31***	0.08
Others’ leave-taking^b^	5.44 (3.01)							0.02	−0.10	−0.05	0.26**	0.32***	0.07
Others’ childcare engagement^b^	4.56 (1.24)								0.11	0.02	−0.10	−0.20*	−0.17^†^
Expected backlash^a^	2.57 (1.82)									−0.13	−0.20*	−0.42***	−0.20*
Expected parental self-efficacy^a^	5.81 (0.90)										0.25**	0.31***	0.18*
Desired parental leave-taking^a^	6.14 (1.56)											0.76***	0.40***
Parental leave-taking intentions^a^	5.58 (1.92)												0.49***
Expected length of parental leave (%)	57.67 (41.77)												

***p < 0.001; **p < 0.01; *p < 0.05; ^†^*p* < 0.10 (all two-tailed). ^a^7-point scale; ^b^9-point scale.

### 3.2 Analytical approach

We first screened the data and checked the statistical assumptions, followed by hierarchical regression analyses conducted separately for the three dependent variables *desired parental leave-taking* (Table 2), *parental leave-taking intentions* (Table 3), and *expected length of parental leave* (Table 4). We used the R package *lavaan* (Rosseel, 2012) for the regression analyses because robust estimation methods are available given assumption violations as well as full information maximum likelihood estimation for treating missing data. Missing data were mainly present for the dependent variables and for predictors related to men’s normative environment (i.e., social support from partners and workplaces and other men’s leave-taking and childcare engagement; 9–13% of missings). Participants with and without missing data did not differ significantly in terms of demographic characteristics (all *p*s > 0.078). Due to the sample size, we do not present more complex models such as multivariate regression or structural equation models. For regression models, interpreting fit indices in *lavaan* is not informative due to the presence of saturated models. In the Supplementary Table S2, we present *F*-tests (which are not available in *lavaan*) for regression models using the R package *lm* (however, accordingly without treatment of missing data and assumption violations).

**Table 2:** Hierarchical regression models (with standardized regression coefficients) for desired parental leave-taking.

	Model 1	Model 2	Model 3	Model 4	Model 5
Step 1: Covariates					
Age	0.07	0.02	−0.00	−0.05	
Country of residence	0.23*	0.26**	0.21**	0.13^†^	0.13
Education level	−0.27**	−0.26**	−0.26**	−0.22**	−0.26**
Relative income	0.07	0.04	0.08	0.08	
Work hours	−0.22^†^	−0.15	−0.13	−0.10	
Step 2: Masculinity and fatherhood beliefs					
Communal prototypes of men		0.26**	0.21*	0.17^†^	0.19^†^
Agentic prototypes of men		−0.08	−0.15^†^	−0.16*	−0.19*
Father role attitudes—childcare		0.11^†^	−0.01	−0.02	
Father role attitudes—breadwinning		−0.13	0.01	0.08	
Step 3: Social support					
Partner support			0.41**	0.42***	0.38**
Workplace support			0.02	−0.06	
Step 4: Additional predictors					
Others’ leave-taking				0.14^†^	0.13^†^
Others’ childcare engagement				−0.09	
Expected backlash				−0.13	
Expected parental self-efficacy				0.15*	0.13^†^
Adjusted *R*^2^	0.12	0.19	0.30	0.35	0.35
*R*^2^ change		0.07	0.11	0.05	

***p < 0.001, **p < 0.01, *p < 0.05, ^†^*p* < 0.10.

**Table 3:** Hierarchical regression models (with standardized regression coefficients) for parental leave-taking intentions.

	Model 1	Model 2	Model 3	Model 4	Model 5
Step 1: Covariates					
Age	0.17*	0.13^†^	0.10^†^	0.05	
Country of residence	0.26**	0.27**	0.21**	0.08	
Educational level	−0.26**	−0.26**	−0.24**	−0.16*	−0.14*
Relative income	0.09	0.05	0.10	0.10	
Work hours	−0.19*	−0.16*	−0.13	−0.08	
Step 2: Masculinity and fatherhood beliefs					
Communal prototypes of men		0.14	0.09	0.02	
Agentic prototypes of men		−0.01	−0.06	−0.06	
Father role attitudes—childcare		0.14^†^	0.05	0.03	
Father role attitudes—breadwinning		−0.23*	−0.11	0.03	
Step 3: Social support					
Partner support			0.32**	0.31**	0.30***
Workplace support			0.11	−0.03	
Step 4: Additional predictors					
Others’ leave-taking				0.24**	0.27***
Others’ childcare engagement				−0.21**	−0.20**
Expected backlash				−0.25**	−0.28***
Expected parental self-efficacy				0.21**	0.22**
Adjusted *R*^2^	0.14	0.21	0.30	0.46	0.47
*R*^2^ change		0.07	0.09	0.16	

***p < 0.001, **p < 0.01, *p < 0.05, ^†^*p* < 0.10.

**Table 4:** Hierarchical regression models (with standardized regression coefficients) for expected length of parental leave in percent of available leave.

	Model 1	Model 2	Model 3	Model 4	Model 5
Step 1: Covariates					
Age	0.18*	0.13	0.13	0.09	
Country of residence	−0.23**	−0.23**	−0.28**	−0.37***	−0.33***
Educational level	−0.09	−0.10	−0.09	−0.06	
Relative income	0.09	0.04	0.07	0.08	
Work hours	−0.21**	−0.17*	−0.15*	−0.14^†^	−0.22**
Step 2: Masculinity and fatherhood beliefs					
Communal prototypes of men		0.06	0.03	−0.00	
Agentic prototypes of men		−0.07	−0.10	−0.12	
Father role attitudes—childcare		0.09	0.02	0.01	
Father role attitudes—breadwinning		−0.24**	−0.15	−0.05	
Step 3: Social support					
Partner support			0.25**	0.25**	0.28***
Workplace support			0.02	−0.03	
Step 4: Additional predictors					
Others’ leave-taking				0.14	
Others’ childcare engagement				−0.18*	−0.22**
Expected backlash				−0.07	
Expected parental self-efficacy				0.14^†^	0.14*
Adjusted *R*^2^	0.13	0.18	0.22	0.27	0.25
*R*^2^ change		0.05	0.04	0.05	

***p < 0.001, **p < 0.01, *p < 0.05, ^†^*p* < 0.10.

In the first set of models (Models 1), we included the covariates age, country of residence (dummy-coded with 1 = Germany and 0 = Belgium), educational level (dummy-coded with 1 = university education or higher and 0 = below university education to reduce number of predictors), relative income, and weekly work hours. We decided on these covariates before data analyses due to prior evidence for relations to men’s parental leave-taking (e.g., Trappe, 2013a, 2013b; Stertz et al., 2017; Geisler and Kreyenfeld, 2019; Marynissen et al., 2019). In the second set of models (Models 2), we added beliefs regarding masculinity and fatherhood, namely communal and agentic prototypes of men, and father role attitudes regarding childcare and breadwinning. In the third set of models (Models 3), we added the social support men received from their partners and their workplace for taking parental leave, and in a fourth step (Models 4), additional predictors related to men’s intended leave-taking for which we did not generate hypotheses (others’ leave-taking, others’ childcare engagement, expected backlash for leave-taking, expected parental self-efficacy). Lastly, we present parsimonious models (Models 5) with only those predictors included that were significant (or tended to be) in Models 4.

### 3.3 Covariates

The covariates explained 12% of variance in desired parental leave-taking, 14% in parental leave-taking intentions, and 13% in the expected length of parental leave (Models 1). Age only emerged as a significant predictor of intended leave-taking in some models, but if so, older age was associated with higher intended leave-taking. Residing in Germany was associated with a higher desire and intention to take leave (but these relations did not hold in later models). In contrast, Belgian residence was related to planning to take a higher percentage of available leave, possibly because the available leave is shorter than in Germany (average expected absolute leave lengths were 10 out of 16 weeks in Belgium, *M* = 10.09, *SD* = 6.63, and four and a half out of 12 months in Germany, *M* = 4.48, *SD* = 4.45). A higher educational level was negatively related to men’s desired parental leave-taking and parental leave-taking intentions. Men’s income relative to their partners was not significantly related to their intended leave-taking. Lastly, longer weekly work hours were related to men expecting to take shorter percentages of parental leave (and in Models 1 and 2 also to lower intentions to take leave).

### 3.4 Hypothesis tests

We found partial support for Hypothesis 1.1, that men’s beliefs that an ideal man has communal attributes would be related to higher intended leave-taking (operationalized in the present research as desired parental leave-taking, parental leave-taking intentions, and expected length of parental leave). Communal prototypes of men were positively related to men’s desired parental leave-taking but not to any other dependent variable. Also, relations were weaker with increasing numbers of predictors, possibly due to correlations amongst predictors (see Table 1). Hypothesis 1.2 postulated that men’s beliefs that an ideal man should have agentic attributes would be related to lower intended leave-taking. We again found support for desired parental leave-taking but none of the other operationalizations of intended leave-taking. Thus, the degree to which men think an ideal man should have agentic attributes was negatively related to their wish to take parental leave. In contrast to communal prototypes of men, relations were stronger in later models.

We did not find support for Hypothesis 2.1, that father role attitudes regarding childcare would be positively related to men’s intended leave-taking. For father role attitudes regarding breadwinning (H2.2), we found significant negative relations in Models 2 between father role attitudes regarding breadwinning on the one side and parental leave-taking intentions as well as the expected length of parental leave on the other, indicating that the more men think it is a father’s role to be involved in breadwinning, the lower their intentions and expected length of parental leave. These relations did not hold when additional, partly correlated (see Table 1) predictors such as social support were added. Yet, only *perceived* support was measured, and men could perceive more or less support from their partner or people at work depending on their father role attitudes. Hence, we possibly did not find support for Hypothesis 2.2 in later models due to correlated measures or even mediation effects.

Lastly, we examined whether the support men perceive to receive from their partners and people at work for taking parental leave was related to their intended leave-taking (H3.1 and 3.2). Across dependent variables and models, support from the partner was a significant predictor, supporting Hypothesis 3.1. The more support for their leave-taking men perceived receiving from their partners, the more they desired to take leave, the more they intended to take leave, and the longer they expected to take leave. In contrast and contradicting Hypothesis 3.2, the support men perceived from people at work was not significantly related to their intended leave-taking. Yet, examining bivariate correlations revealed that partner support and workplace support were significantly correlated (see Table 1). Apparently, perceiving much support from the partner was positively related to perceiving much support from people at work for the expectant fathers in our sample. This could, on the one hand, suggest a selection effect (i.e., one also selects the places where one works and continues to work as fitting) or, on the other hand, wishful thinking of the care-oriented fathers to receive support, generalized to the social environment.

### 3.5 Robustness checks and exploratory analyses

As a robustness check for the partner support findings, we ran additional analyses in which we controlled for men’s perception of their partner’s prototypes of men and father role attitudes (see Supplementary Table S3). Including these measures did not affect the results for partner support on men’s intended leave-taking (*β*s = 0.26–0.40), suggesting that active support or discouragement from partners plays a role for men’s intended leave-taking beyond the partner’s general gender egalitarianism. Moreover, we repeated the analyses for the expected length of parental leave, now also controlling for whether participants intended to take leave part-time or full-time (see Supplementary Table S4). For that, we excluded participants from the analyses who did not intend to take any leave and added a dummy variable for part-time versus full-time leave-takers. This exclusion reduced the sample size to 107, but the results of hypotheses tests were not affected. Still, the support men perceived from their partners for taking leave was the main robust predictor of their expected length of parental leave (*β* = 0.29, *p* = 0.007).

As exploratory analyses, we examined further predictors that could be related to men’s intended leave-taking based on past research: other men’s leave-taking in their personal environment, other men’s childcare engagement, expected backlash for leave-taking, and expected parental self-efficacy (see Tables 2–4, Models 4). For all dependent variables, we found small positive relations with men’s expected parental self-efficacy: The more men expected to be capable of taking care of their child in the future, the more they wished and intended to take leave and the longer they expected to take leave. Counterintuitively, how much other men engaged in childcare was negatively related to men’s parental leave-taking intentions and expected length of parental leave. Thus, the less men perceived other men to be engaged in childcare, the more and the longer they intended to take leave (or perhaps: the more and the longer the participants intended to take leave, the less they perceived other men to be engaged in childcare – suggesting a contrast effect). Others’ leave-taking and expected backlash for leave-taking were additionally related to men’s parental leave-taking intentions: The more other men took leave before them, and the less they expected backlash for leave-taking, the higher were men’s intentions to take parental leave.

However, the models including exploratory predictors were rather complex given the sample size and could be prone to overfitting and lack of generalizability to other datasets. Therefore, we aimed to check whether the predictors that appeared relevant for intended leave-taking in the larger models also hold in more parsimonious models (Models 5) including only predictors that were significant in Models 4 or showed trends. For desired parental leave-taking, especially the support men receive from their partners for leave-taking seemed to be related to their wish to take leave. In addition, we found a small relation between agentic prototypes of men and desired parental leave-taking, suggesting that the less men saw an ideal man as agentic, the more they wished to take parental leave. Communal prototypes of men and the expected parental self-efficacy were not significantly related to desired parental leave-taking in the parsimonious model. Overall, these predictors, including covariates, explained 35% of variance in desired parental leave-taking. For parental leave-taking intentions, again, partner support emerged as an important predictor with a medium-sized relation, besides small relations for others’ leave-taking, others’ childcare engagement, expected backlash for leave-taking, and expected parental self-efficacy beliefs. We were able to explain the largest amount of variance in parental leave-taking intentions (47% of variance explained). Lastly, the support men perceived receiving from their partners for taking leave, how much other men in their personal environment engaged in childcare, and their expected parental self-efficacy were also predictive of the percentage of parental leave men expected to take. For this more behavior-oriented dependent variable, we were able to explain 25% of variance in the parsimonious model.

## 4 Discussion

Parental leave has been discussed as a tool to foster men’s engagement in communal roles with benefits for men themselves as well as their personal environment. However, men continue to take less parental leave than their partners, raising the question of how their intentions to take parental leave are shaped. In the current paper, we investigated predictors of men’s intended parental leave-taking before birth, using data from soon-to-be fathers in Belgium and Germany. To gain a deeper understanding of men’s intended leave-taking, we examined different operationalizations on a continuum of behavioral preferences to more concrete behavioral intentions.

The findings provide support for the hypothesized positive relation between partner support and men’s intended leave-taking (H3.1). The more support men perceived from their partners to take parental leave, the more they desired to take leave, intended to do so, and aimed to take a higher percentage of available leave. We additionally found partial support for the expected negative relation of agentic prototypes of men and men’s intended leave-taking (H1.2) and, to a lesser degree, for the expected positive relation of communal prototypes of men and men’s intended leave-taking (H1.1). That is, the more men thought an ideal man has agentic attributes (e.g., being independent or assertive) the less they wished to take parental leave. Seeing an ideal man as communal (e.g., communicative or emotional) tended to be related to a stronger wish to take parental leave. Yet, we did not find any significant relations of prototypes with other operationalizations of men’s intended leave-taking besides their wish to take leave. Moreover, the results provided partial support for the hypothesized relation of father role attitudes regarding breadwinning and intended leave-taking (H2.2). Men with more breadwinning-oriented father role attitudes partially intended less to take leave and a lower percentage of the available leave. Father role attitudes regarding childcare and perceived workplace support for leave-taking were not related to men’s intended leave-taking, providing no support for Hypotheses 2.1 and 3.2.

However, exploratory analyses suggested that men’s parental leave-taking intentions were also predicted by other men’s engagement in childcare and their take-up of parental leave, the backlash participants expected to receive for taking parental leave, and participants’ expected self-efficacy as a parent and caregiver. Moreover, how much other men engaged in childcare was also negatively related to how long men expected to take leave. Lastly, the more capable men felt of taking care of their child in the future (i.e., their expected parental self-efficacy), the longer they expected to take leave.

The perceived support men receive from their partners for taking parental leave played a crucial role in their intended leave-taking in the current study. This finding suggests that parental leave decisions are shaped through negotiations in partnerships. As the transition to parenthood is often experienced as a couple, the new life tasks have to be negotiated and distributed interpersonally. Qualitative research on men’s leave-taking has focused on the decision-making process of couples who shared parental leave before, concluding that often only limited negotiations were taking place (Beglaubter, 2017). Even when men desired to take leave, decisions were often based on a strong sense of mothers’ entitlement for leave-taking, which placed fathers’ leave-taking as a “bonus” to the mothers’ share. Nevertheless, within these boundaries, the female partners’ point of view remained an important driver for determining parental leave shares, for example, when partners wanted to return to work soon or were not eligible to take leave. Brandt (2017) also discussed men’s leave-taking as a matter of negotiation in partnerships. However, there the negotiation process was examined implicitly by looking at distributions of economic resources in partnerships, working conditions of partners, and gendered values, suggesting, for example, that partners’ family orientation hinders, whereas fathers’ family orientation helps their take-up of leave. While the role of economic considerations or gender ideologies has thus been discussed before, the current paper goes one step further in showing that partners’ active support or discouragement can contribute to men’s intended leave-taking beyond relative income shares or gender role attitudes. Even though this provides a tangible parameter for influencing men’s leave-taking (i.e., partners’ active encouragement), the conclusion of the current findings should not solely be that the responsibility for men’s leave-taking lies with their partners. This would make women responsible for yet another aspect and add to the pressures on women when combining family and career and facing intensive motherhood norms (e.g., Meeussen and Van Laar, 2018). Nevertheless, mothers can play a key role, functioning as gatekeepers for men’s leave-taking, especially in the case of transferable leave periods between partners (Allen and Hawkins, 1999; Cannito, 2020). Thus, the perceived role of partners for men’s leave-taking is crucial given specific policy designs, but decision-making processes remain a joint task for couples in which women *and* men carry responsibility.

Besides partner support for leave-taking, no other variable was consistently related to all operationalizations of men’s intended leave-taking. This suggests that different predictors may be relevant for men’s leave-taking the more concrete their intentions become. Men’s conception of an ideal, prototypical man (especially in terms of agency) was related to their desire to take parental leave but not to the more behavior-oriented operationalizations of intended leave-taking, such as their expected length of leave. It is intuitive that prototypes of men as more abstract masculinity ideals are relevant for shaping behavioral preferences because they prescribe what is desirable for group members (Oakes et al., 1998; Wenzel et al., 2007; Hogg et al., 2012). Yet, when looking at more behavior-oriented outcomes, reality constraints are introduced, which require going beyond behavioral preferences based on ideal circumstances. As found in the current paper, outside influences and men’s broader normative environment (e.g., how much other men before them engaged in leave-taking and childcare, or the negative consequences men expect to face for wanting to take leave) additionally contribute to their concrete intentions for taking parental leave. Also, men’s expected parental self-efficacy, as the degree to which they perceived themselves as *able* to take care of their child independently, provides a reality check and was found to be related to how long men planned to take leave in the current study. Still, explaining correlates of more concrete leave-taking plans remained more difficult, and we were able to explain the smallest amount of variance in men’s expected length of parental leave (*R*^2^_adj_ = 0.25 compared to 0.35 for desired leave-taking and 0.47 for leave-taking intentions), in line with general models of attitudes, behavioral intentions, and behavior (Ajzen, 1991). Likely, the specific length of the planned leave depends more strongly on individual circumstances within the relationship and external reality constraints than behavioral preferences or intentions do.

Besides masculinity ideals, we also included father role attitudes, but results were mixed and only significant in a few models in line with hypotheses. An explanation for that could be a self-selection process within our sample: Highly identified expectant fathers, who may relate to current norms of involved fatherhood, could have been more motivated to participate in the study than traditional, work-focused expectant fathers. The general high orientation towards care (i.e., high ratings on childcare-related father role attitudes and intended leave-taking) underline this assumption, making it more difficult to find significant relations due to restricted variance. In a more diverse sample, internal contributors such as attitudes towards fatherhood likely are more relevant next to external influences like social support. Moreover, in a similar study on predictors of men’s leave-taking in the US, only maternal essentialism emerged as a correlate of men’s leave-taking in contrast to parenting role beliefs (a similar measure to our father role attitudes; Berrigan et al., 2021). Thus, whether men think women are *naturally* better caregivers could be more closely related to childcare decisions regarding newborns than more general parenting beliefs. This is in line with evidence on the relevance of breastfeeding for parental leave-taking decisions (Beglaubter, 2017; Bueno and Grau-Grau, 2021). A strong endorsement of breastfeeding puts mothers in the role of primary caregivers and reduces men’s claim for taking parental leave because of biological differences. Hence, future research should examine more closely how essentialist, compared to general beliefs toward parenting roles, are related to men’s leave-taking, using more representative samples.

Furthermore, we did not find evidence for the relation between workplace support and men’s intended leave-taking. This contrasts with past research that stresses the importance of the workplace for men’s leave-taking decisions (Bygren and Duvander, 2006; Kaufman and Almqvist, 2017; Brandth and Kvande, 2019; Haas and Hwang, 2019). However, other studies also failed to find consistent relations for men’s higher workplace support as compared to their partner (Brandt, 2017) or for supervisor support with men’s leave-taking (whereas workgroup support and workplace norms were related to men’s leave-taking; Haas et al., 2002; Samtleben et al., 2019a). The latter finding suggests that, in future research, workplace support should be measured separately for colleagues and supervisors instead of using a combined measure like in the current study. Moreover, participants could have selected their workplace partly based on correspondence with their personal values, such as family orientation, reducing the relevance of workplace support for predicting men’s intended leave-taking. In addition, workplace support was correlated with other predictors in the models, namely others’ leave-taking and expected backlash effects. When asking expecting fathers how much other men in their personal environment took leave, colleagues are likely an important reference group. Moreover, being encouraged or discouraged by people at work signals whether men could expect negative consequences and backlash for taking leave. Future longitudinal research could therefore shed light on the interplay and temporal order of these constructs and how they contribute to men’s leave-taking decisions. In addition, some participants commented that they filled in the survey earlier than 3 months before birth and had not made concrete plans regarding parental leave yet. Possibly, conversations with people at work take place at later stages in men’s decision-making process, and there had not been much room for receiving support from the workplace yet.

In addition to hypotheses tests, we explored further predictors of men’s intended leave-taking. Results confirmed the relevance of fearing backlash (e.g., Vogt and Pull, 2010; Samtleben et al., 2019a): The more men expected negative consequences when taking leave, the less they intended to take leave. Furthermore, these explorations yielded additional evidence for how men’s leave-taking decision appears to be shaped within a normative environment and how others’ behavior is related to their own intentions. Here, other men can function as role models who show the feasibility of taking leave as a man, for example, by reducing the perception of external barriers (Morgenroth et al., 2015). In fact, backlash effects and career consequences following men’s leave-taking are often less negative than expected (Fleischmann and Sieverding, 2015; Samtleben et al., 2019a; see also mixed evidence in the review by Steffens et al., 2019). Moreover, seeing other men take leave can reduce self-stereotyping and facilitate the consideration of counter-stereotypic engagement – which parental leave-taking traditionally is for men (Morgenroth et al., 2015; also see Asgari et al., 2010). Lastly, role modeling is especially effective in the case of similarity and shared group membership, speaking again to the inspirational role of male colleagues’ leave-taking (Bygren and Duvander, 2006). Whereas we found this motivational relation of other men’s leave-taking with participants’ leave-taking intentions, other men’s childcare engagement was negatively related to participants’ leave-taking intentions and expected length of parental leave. It is possible that other men who engage less in childcare than their partners function as negative role models (see Lockwood et al., 2002), showing men what they would miss out on. Alternatively, given the correlational data and unclear causal order, men with stronger leave-taking intentions could perceive other men as engaging comparatively little in childcare. Lastly, the negative relation could also be interpreted inversely as perceiving other men to be highly engaged in childcare being related to lower leave-taking intentions. In fact, men who do more childcare than their partners, like in the case of stay-at-home dads, indeed often experience backlash (Steffens et al., 2019), which could deflate men’s leave-taking intentions.

### 4.1 Strengths and limitations

The current results should be viewed in light of the following limitations. Most importantly, we report on cross-sectional correlational data and are therefore not able to draw causal conclusions about precursors of men’s intended leave-taking. Although experimental designs allow for such conclusions, they can be ethically questionable and difficult to implement for life decisions such as parenthood and parental leave-taking (for experimental evidence for hypothetical leave-taking, see Rudman and Mescher, 2013; Scheifele et al., 2021). The current study adds to existing research by examining intentions of men who are actually becoming parents and are facing parental leave-taking decisions. Naturally, an interesting avenue for future research is to gain more insight into predictors of men’s actual leave-taking instead of mere intentions. Still, by zooming in on men’s intended leave-taking and different nuances from preferences to more concrete plans, we gain a deeper understanding of which factors are related to men’s leave-taking decisions before birth. In addition, analyzing cross-sectional data on men’s leave-taking intentions enables us to make better predictions for a longitudinal assessment of men’s leave-taking decisions across the transition to parenthood.

Although the current study goes beyond student samples, we still rely on a convenience sample with limited representativeness in terms of socio-economic status or gender and parenting attitudes. Therefore, the current findings cannot easily be generalized to the population of expectant fathers in Belgium and Germany. Nevertheless, one could argue that it is particularly interesting and a more conservative test to look at how, for this sample, leave-taking intentions are shaped through attitudes and normative environments because external factors such as whether parents can financially afford men’s leave-taking play a minor role here. Also, if there is limited variance in our sample, the correlations we found likely are lower boundaries of true correlations in more diverse samples, including more traditional fathers.

Another limitation can be found in the start of the data collection at the end of 2021 when the global COVID-19 pandemic was ongoing. However, only few participants completed the surveys when measures such as mandatory teleworking were still implemented. In addition, although the pandemic had consequences for parents’ division of labor, with men increasing their time spent at home, mothers continued to shoulder the majority of childcare and housework (Yerkes et al., 2020; Hipp and Bünning, 2021; Kreyenfeld and Zinn, 2021; Petts et al., 2023; Van Tienoven et al., 2023; research conducted in Belgium, Germany, the Netherlands, the United Kingdom, Canada, and the United States). Researchers in Belgium concluded that changes in the division of household labor were rather temporal and that the inertia of gender roles is still evident (Van Tienoven et al., 2023). Thus, while the unique period in which parts of the data were collected should be considered, we do not think that the current findings are caused by this period but likely generalize to other periods as well.

Methodologically, we used several non-validated measures due to a lack of validated alternatives, resulting in issues with internal consistencies and ceiling effects. Lastly, we did not reach the required sample size based on an *a-priori* power analysis. As a result, we were not able to detect small effects and, at times, only found trends in the data. Moreover, sample sizes varied across countries of data collection which could lead to biased estimates and impeded cross-national comparisons. Such examinations would have been interesting though based on the differing results of country of residence across dependent variables, speaking to the role of policy design for men’s intended leave-taking. We, therefore, encourage future longitudinal studies on the relations between men’s parental leave-taking intentions and actual leave-taking, including larger, more representative samples and validated measures.

### 4.2 Conclusion

We see the contribution of the present research in gaining first insight into the parental leave-taking intentions of expectant fathers while addressing different facets of the studied constructs and carving out the role that men’s social setting plays in their orientation towards care. Across analyses, higher levels of partner support were accompanied by a higher desire and intention of expectant fathers to take (longer) leave, illustrating the role of partners as gatekeepers for men’s leave-taking. Other predictors were more relevant for different facets of intended leave-taking, speaking to a nuanced assessment of such. Notions of what it means to be a man tended to be linked to whether expectant fathers wished to take parental leave, whereas men’s broader normative environment was especially predictive of their behavioral intentions to take leave. Taken together, these findings advance current knowledge on predictors of men’s intended parental leave uptake but also of men’s involvement in childcare more generally, as parental leave can represent a gateway for continuous father involvement.

## Data availability statement

The original contributions presented in the study are publicly available. The raw data omitting demographic information can be found here: https://osf.io/f7jeh/.

## Ethics statement

The studies involving humans were approved by the University of Leuven’s Social and Societal Ethics Committee. The studies were conducted in accordance with the local legislation and institutional requirements. Written informed consent for participation was not required from the participants or the participants’ legal guardians/next of kin because the study was fully conducted online and therefore participants provided informed consent digitally as was specified in the ethics application.

## Author contributions

CS, CL, and MCS contributed to the conception and design of the study. CS spearheaded data collection, performed the statistical analyses, and wrote the first draft of the manuscript. All authors contributed to manuscript revision, read, and approved the submitted version.

## Footnotes

^1^https://aspredicted.org/3HY_17Q

^2^Data are part of an ongoing longitudinal study on men’s parental leave-taking with data having been collected at roughly 3 months before birth, and planned measurement points at 4 months after birth and 12 months after birth. As the current study focuses on men’s leave-taking intentions before birth and data collection for later measurement points is ongoing, we only present analyses on the data collected before birth.

^3^A paternity leave of an additional 20 days (15 days until 2022) is available for fathers only (FOD, 2023). As no equivalent exists for Germany and because of ceiling effects in our data for the intended uptake (almost all fathers intend to take the full amount), we do not present results for paternity leave.
