# Supplementary material for: Predictors of expectant fathers’ parental leave-taking intentions before birth: masculinity, fatherhood beliefs, and social support
Source: Front Psychol. 2024 Feb 12;15:1247193. doi: 10.3389/fpsyg.2024.1247193 (PMC10895060; doi:10.3389/fpsyg.2024.1247193)
Supplement: Supplementary file 1 [file Table_1.DOCX]

# *Supplementary material*

# Predictors of expectant fathers’ parental leave-taking intentions before birth: Masculinity, fatherhood beliefs, and social support

Carolin Scheifele^*^, Colette Van Laar, Melanie C. Steffens

***Correspondence**:

Carolin Scheifele

[carolin.scheifele@rptu.de](mailto:carolin.scheifele@rptu.de)

# Factor analyses

We conducted exploratory factor analyses (EFAs) to examine the underlying factor structure of the measures, given the fact that we adapted previously published scales and the issues we had with internal consistency for some. We determined the appropriate number of factors by means of parallel analysis and used the maximum likelihood estimation method with an oblimin rotation in the factor analyses.

For the agency subscale of the prototypes of men measure, a one-factor-solution was sufficient (TLI = .97, RMSEA = .055), although one factor loading was rather weak (.39, all others > .59). For the communion subscale, parallel analysis suggested a two-factor-solution; however, the indices indicated some degree of overfitting (TLI = 1.02, RMSEA = 0.00). We, therefore, also aggregated the items to an overall communion scale for a number of reasons: a) all factor loadings for the one-factor model were above .46, b) the internal consistency was adequate as indicated by a Cronbach’s alpha of .77, c) we did not want to raise the number of predictors in the main analyses further given the sample size, and d) the one-factor-solution also had a similar fit to the more fine-grained approach in the original scale (Hentschel et al., 2019).

For the caregiving subscale of the father role attitudes measure, also a two-factor solution was suggested but model fit was not good (TLI = .75, RMSEA = .11). We, therefore, only aggregated the items loading on the first factor to form a scale that also captured general caregiving father role attitudes best in our opinion (factor loadings of aggregated items: .46 – 1.00, all others < .25). We did not include the other items in the main analyses as only one item had a factor loading above .40 for the second factor and since they tapped into less central aspects of the caregiving attitudes (e.g., fathers’ caregiving as helping out their partners or job interference with childcare). For the breadwinning subscale, a one-factor solution had good model fit (TLI = .99, RMSEA = .04, factor loadings = .37 – .72). In sum, however, the psychometric properties of the scales represent a limitation of the study as we mention in the discussion section in the main article.

# Further robustness checks

## Including outliers

We ran the models including all predictors again with outliers included. This led to some changes regarding the results for desired parental leave-taking. Instead of a trend for communal prototypes of men, we now found a significant relation to men’s desired parental leave-taking (β = .18, *p* = .043), whereas agentic prototypes of men were not significantly related anymore (β = -.10, *p* = .208). In terms of the additional predictors, others’ leave-taking was not a significant predictor of men’s desired leave-taking anymore (β = .10, *p* = .133) but instead expected backlash was (β = -.17, *p* = .034). The results for parental leave-taking intentions did not change substantially when outliers were included. For the expected length of parental leave, perceived self-efficacy was not significantly related anymore (β = .10, *p* = .106). Taken together, some relations were weakened and, thus, power could be too low to detect these smaller relations. However, none of the coefficients changed direction, and the results including outliers would not substantially affect the conclusions drawn.

## Absolute parental leave length

As we did not preregister using a percentage measure for the expected parental leave length, we checked how using absolute expected leave lengths would affect the results in the fourth and fifth model. Agentic prototypes were significantly related to men’s expected length of parental leave, suggesting that seeing an ideal man as agentic was related to a lower expected length of leave, in line with H1.2 (Model 4: β = -.26, *p* = .028, Model 5: β = -.25, *p* = .019). However, parental self-efficacy did not emerge as a significant predictor of expected leave length anymore (Model 4: β = .09, *p* = .264, Model 5: β = .06, *p* = .485). Moreover, we only found a trend for others’ childcare engagement in Model 5 (β = -.17, *p* = .074).

## Excluding sociability items from measure for communal prototypes of men

As preregistered, we excluded the items *communicative* and *likeable* from the communion subscale used to measure prototypes of men. In past research, no differences between men and women were found in terms of sociability (Hentschel et al., 2019), which could result in ceiling effects. However, the mean was only slightly lower when these items were excluded, *M* = 4.98, *SD* = 0.83 (with items included: *M* = 5.10, *SD* = 0.79). Using this alternative scale led to a weaker trend for communal prototypes of men and men’s desired parental leave-taking (Model 4: β = .12, *p* = .148, Model 5: β = .15, *p* = .099). Also, the relation of agentic prototypes of men and men’s desired parental leave-taking was weaker (Model 4: β = -.15, *p* = .068, Model 5: β = -.18, *p* = .040). Results for parental leave-taking intentions or the expected length of leave were not affected.

# Deviations from preregistration and additional measures

We conducted an a-priori power analysis before collecting data but were not able to recruit the required sample size of *N* = 188 (*N* = 200 including a buffer) despite continuous long term recruitment efforts. In the manuscript, we present a sensitivity analysis to indicate which effect sizes we were able to detect given the final sample size of *N* = 143.

We preregistered that, for the T1 data, we would conduct regression analyses to examine the relations between male prototypes, possible selves, and parental leave-taking intentions (see preregistration for details on measures, <https://aspredicted.org/3HY_17Q>). As not included in the manuscript, we present findings for the relations of male prototypes (named prototypes of men in the following), possible selves, and intended parental leave-taking here. Possible task engagement was assessed on a scale from 1 = “I will do this exclusively” to 7 = “My partner will do this exclusively”, but the scale was reverse coded during data analyses to facilitate interpretation. We hypothesized that more communal prototypes of men and more communal possible selves before birth are positively related to men’s parental leave-taking intentions. Results of regression analyses are presented in Table S5. In fact, communal prototypes of men were positively related to men’s desired parental leave-taking and tended to be positively related to men’s parental leave-taking intentions. We did not find significant relations for men’s communal possible self-concept or possible task engagement regarding childcare. However, possible task engagement regarding work emerged as a predictor of men’s expected absolute length of leave. The more men thought they will spend time on their paid job in the future as compared to their partners, the shorter they planned to take leave. For the percentage measure, we also found a trend in this direction. Moreover, men’s agentic possible self-concept tended to be negatively related to men’s parental leave-taking intentions. The more men thought that agentic attributes will describe them in the future, the lower their parental leave-taking intentions tended to be.

We further planned to conduct analyses for men’s intended *paternal* leave-taking besides their parental leave-taking. As paternal leave is only offered in Belgium (before 2023: 15 days, since 2023: 20 days), we conducted the analyses with Belgian participants (*N* = 106) and did not include country of origin as a covariate (see Table S6). Descriptively, participants had a strong desire (*M* = 6.73, *SD* = 0.92, assessed on 7-point scale) and intention (*M* = 6.67, *SD* = 0.97, assessed on 7-point scale) to take paternal leave. They planned to take on average 14.47 days of paternal leave (*SD* = 2.29). In line with the preregistered hypothesis, communal prototypes of men were positively related to men’s expected length of paternal leave, suggesting that the more men saw an ideal man as communal, the longer they intended to take paternal leave. In addition, we found a positive relation between men’s communal possible self-concept and their desire to take paternal leave: The more men expected communal attributes to describe them in the future, the more they wished to take paternal leave. Exploratory analyses suggest a positive relation for men’s possible task engagement regarding routine household tasks and their expected length of paternal leave. The more men expected to engage in routine household tasks as compared to their partners, the longer they expected to take paternal leave. Moreover, we found trends for negative relations between men’s agentic possible self-concept and their paternal leave-taking intentions as well as men’s possible task engagement regarding non-routine household tasks and their desired paternal leave-taking. Lastly, a positive trend emerged for men’s possible task engagement regarding work and their desired paternal leave-taking. Contrary to expectations, men who expected to work more as compared to their partners had a stronger desire to take paternal leave.

Additional measures that are not part of this manuscript were others’ support for leave-taking (e.g., friends, family), others’ prototypes of men, others’ father role attitudes, desired possible selves (attributes and tasks), timepoint of leave-taking, expected job consequences of leave-taking, reasons for or against leave-taking, partner’s leave-taking plans, gender identification, father identification, career vs. family orientation, and gender role attitudes. We explored whether some of these measures were predictive of men’s intended leave-taking but arrived at the set of additional predictors that is currently presented in the analyses as the most parsimonious selection. Moreover, only partial data were available for some measures (e.g., others’ support for leave-taking or partner’s leave-taking plans), which would have reduced the sample size substantially.

# Supplementary Tables

**Table S1**. Descriptive statistics separated by country

|  | *M*_Belgium_ (*SD*) | *M*_Germany_ (*SD*) |  | *Correlations* (*N*_Belgium_ = 89 – 115, above diagonal, *N*_Germany_ = 26 – 28, below diagonal) | | | | | | | | | | | |
| --- | --- | --- | --- | --- | --- | --- | --- | --- | --- | --- | --- | --- | --- | --- | --- |
|  |  |  | 1. | 2. | 3. | 4. | 5. | 6. | 7. | 8. | 9. | 10. | 11. | 12. | 13. |
| 1. Prototypes of men – Communion | 5.15  (0.80) | 4.89 (0.69) |  | .27** | .15 | .00 | .16 | .13 | .18^†^ | .04 | -.04 | .23* | .35*** | .23* | .10 |
| 1. Prototypes of men – Agency | 5.22 (0.79) | 5.16 (0.96) | .03 |  | -.01 | .19* | .15 | .11 | -.05 | -.03 | .06 | .04 | -.05 | -.06 | -.10 |
| 1. Father role attitudes – Childcare | 8.18 (0.99) | 8.39 (0.72) | .08 | -.13 |  | -.10 | .33*** | .02 | -.06 | -.03 | -.07 | .12 | .15 | .13 | .09 |
| 1. Father role attitudes – Breadwinning | 4.49 (1.46) | 4.32 (1.79) | .33^†^ | .31 | -.09 |  | -.27** | -.05 | -.18^†^ | .18^†^ | .12 | -.20* | -.06 | -.22* | -.29** |
| 1. Partner support | 7.75 (1.45) | 8.43 (1.57) | .06 | .00 | .12 | -.42* |  | .36*** | .19^†^ | -.02 | -.07 | .18^†^ | .36*** | .39*** | .33*** |
| 1. Workplace support | 6.18 (1.72) | 7.04 (1.77) | .54** | -.13 | .29 | .11 | .21 |  | .38*** | -.08 | -.34*** | .13 | .24* | .31** | .17^†^ |
| 1. Others’ leave-taking | 4.96 (3.03) | 7.21 (2.18) | .02 | -.03 | -.06 | -.22 | .19 | .02 |  | .03 | -.13 | -.04 | .27** | .35*** | .24* |
| 1. Others’ childcare engagement | 4.66 (1.14) | 4.18 (1.52) | .10 | .23 | -.08 | .34^†^ | -.20 | .19 | .23 |  | .23* | .07 | .00 | -.24* | -.22* |
| 1. Expected backlash | 2.60 (1.81) | 2.43 (1.89) | -.36^†^ | .25 | -.17 | -.20 | -.09 | -.50** | .04 | -.20 |  | -.15 | -.23* | -.44*** | -.29** |
| 1. Expected parental self-efficacy | 5.78 (0.89) | 5.93 (0.93) | .12 | .21 | .18 | -.09 | .11 | .07 | -.24 | -.12 | .01 |  | .27** | .28** | .23* |
| 1. Desired parental leave-taking | 6.03 (1.60) | 6.54 (1.35) | -.04 | -.01 | .12 | -.45* | .92*** | .16 | .02 | -.37* | -.09 | .15 |  | .77*** | .48*** |
| 1. Parental leave-taking intentions | 5.44 (1.94) | 6.12 (1.79) | -.10 | .05 | .22 | -.23 | .65*** | .20 | -.02 | -.01 | -.36^†^ | .41* | .68*** |  | .59*** |
| 1. Expected length of parental leave | 63.05 (41.45) | 37.35 (37.08) | -.08 | -.40* | .14 | -.26 | .27 | -.03 | -.24 | -.21 | .10 | .06 | .31 | .36^†^ |  |

*Notes.* *** *p* < .001, ** *p* < .01, * *p* < .05, ^†^ *p* < .10 (all two-tailed).

**Table S2.** F-tests for regression models

| Dependent variables | Model 1 | Model 2 | Model 3 | Model 4 | Model 5 |
| --- | --- | --- | --- | --- | --- |
| Desired parental leave-taking | *F*(5, 124) = 4.79, *p* < .001 | *F*(9, 120) = 3.83, *p* < .001 | *F*(11, 118) = 5.71, *p* < .001 | *F*(15, 113) = 5.33, *p* < .001 | *F*(7, 121) = 9.50, *p* < .001 |
| Parental leave-taking intentions | *F*(5, 125) = 5.21, *p* < .001 | *F*(9, 121) = 4.56, *p* < .001 | *F*(11, 119) = 5.97, *p* < .001 | *F*(15, 114) = 7.63, *p* < .001 | *F*(6, 123) = 17.63, *p* < .001 |
| Expected length of parental leave (%) | *F*(5, 120) = 4.91, *p* < .001 | *F*(9, 116) = 4.43, *p* < .001 | *F*(11, 114) = 4.61, *p* < .001 | *F*(15, 109) = 4.47, *p* < .001 | *F*(5, 120) = 9.36, *p* < .001 |

We used the R package *lavaan* in the main analyses for robust estimation methods due to assumption violations and for treating missing data. Therefore, no F-tests were available and also no fit indices because models were saturated. To provide some information on the overall fit of the regression models, we present F-tests for regression models conducted with the R package *lm* (however, without adjustments for assumption violations and with a lower sample size due to missing data).

**Table S3.** Regression models (with standardized regression coefficients) for intended parental leave-taking including perceived partner variables

|  | Desired parental leave-taking | Parental leave-taking intentions | Expected length of parental leave (%) |
| --- | --- | --- | --- |
| Age | -.01 | .09 | .12 |
| Country of residence | .20* | .20** | -.30** |
| Educational level | -.27** | -.27** | -.10 |
| Relative income | .08 | .12 | .10 |
| Work hours | -.11 | -.12 | -.14* |
| Communal prototypes of men | .25** | .15 | .09 |
| Agentic prototypes of men | -.13 | .02 | -.11 |
| Father role attitudes – Childcare | .01 | .08 | .03 |
| Father role attitudes – Breadwinning | -.00 | -.09 | -.13 |
| Partner support | .40** | .31** | .26** |
| Workplace support | .03 | .11 | .02 |
| Partners’ communal prototypes of men^a^ | -.03 | -.10 | -.11 |
| Partners’ agentic prototypes of men^a^ | -.06 | -.17^†^ | .02 |
| Partners’ father role attitudes^b^ | -.12 | -.05 | -.03 |
| Adjusted *R*^2^ | .31 | .31 | .21 |

*Notes.* ^a^ Participants were asked about their partner’s opinion on what it means to be a man using the same communal and agentic attributes as for the main measure concerning their own prototypes of men. ^b^ Participants were asked how their partner sees the responsibility of a father for his child using a single bipolar item ranging from 1 = “Earning money to meet his child’s financial expenses” to 7 = “Physically and socially caring for his child”. *** *p* < .001, ** *p* < .01, * *p* < .05, † *p* < .10.

**Table S4.** Hierarchical regression models (with standardized regression coefficients) for expected length of parental leave including full-time vs. part-time leave as covariate

|  | Model 1 | Model 2 | Model 3 | Model 4 |  | Model 5 |
| --- | --- | --- | --- | --- | --- | --- |
| Age | .11 | .09 | .08 | .05 |  |  |
| Country of residence | -.35** | -.35** | -.38*** | -.42*** |  | -.44*** |
| Educational level | -.07 | -.09 | -.13 | -.14 |  |  |
| Relative income | .10 | .06 | .09 | .10 |  |  |
| Work hours | -.13 | -.11 | -.12^†^ | -.14^†^ |  | -.16* |
| Part-time vs. full-time leave | -.04 | -.04 | -.02 | -.03 |  |  |
| Communal prototypes of men |  | .01 | -.01 | -.01 |  |  |
| Agentic prototypes of men |  | -.07 | -.10 | -.15 |  |  |
| Father role attitudes – Childcare |  | .02 | -.03 | -.03 |  |  |
| Father role attitudes – Breadwinning |  | -.14 | -.07 | -.02 |  |  |
| Partner support |  |  | .27* | .29** |  | .21* |
| Workplace support |  |  | -.12 | -.09 |  |  |
| Others’ leave-taking |  |  |  | .05 |  |  |
| Others’ childcare engagement |  |  |  | -.16^†^ |  | -.16* |
| Expected backlash |  |  |  | .10 |  |  |
| Expected parental self-efficacy |  |  |  | .14 |  |  |
| Adjusted *R*^2^ | .15 | .14 | .18 | .21 |  | .20 |

*Notes.* *** *p* < .001, ** *p* < .01, * *p* < .05, † *p* < .10.

**Table S5.** Preregistered regression models (with standardized regression coefficients) including possible selves as predictors

|  | Desired parental leave-taking | Parental leave-taking intentions | Expected (absolute) length of parental leave | Expected length of parental leave (%) |
| --- | --- | --- | --- | --- |
| Age | .05 | .14* | .04 | .15^†^ |
| Country of residence | .28** | .31*** | .43** | -.15 |
| Educational level | -.30** | -.33*** | -.08 | -.15 |
| Relative income | .07 | .12 | .02 | .08 |
| Work hours | -.14 | -.12 | -.07 | -.13 |
| Communal prototypes of men | .21* | .18^†^ | -.01 | .09 |
| Agentic prototypes of men | -.08 | -.00 | -.19^†^ | -.10 |
| Communal possible self-concept | .01 | -.07 | -.02 | -.09 |
| Agentic possible self-concept | -.03 | -.14^†^ | -.02 | -.05 |
| Possible task engagement – Childcare | -.16 | -.13 | -.17 | -.14 |
| Possible task engagement – Work | -.12 | -.01 | -.34** | -.14^†^ |
| Possible task engagement – Routine household tasks | .06 | .07 | -.02 | -.09 |
| Possible task engagement – Non-routine household tasks | -.06 | -.11 | -.06 | -.15 |
| Adjusted *R*^2^ | .14 | .18 | .20 | .17 |

*Notes.* *** *p* < .001, ** *p* < .01, * *p* < .05, † *p* < .10.

**Table S6.** Preregistered regression models (with standardized regression coefficients) for men’s intended *paternal* leave-taking

|  | Desired paternal leave-taking | Paternal leave-taking intentions | Expected (absolute) length of paternal leave |
| --- | --- | --- | --- |
| Age | .14 | .08 | .15* |
| Educational level | -.05 | .05 | .08 |
| Relative income | .01 | -.12 | -.15 |
| Work hours | -.11 | .12 | -.04 |
| Communal prototypes of men | -.15 | .15 | .24* |
| Agentic prototypes of men | .06 | .03 | -.07 |
| Communal possible self-concept | .32* | -.02 | -.02 |
| Agentic possible self-concept | -.15 | -.19^†^ | -.14 |
| Possible task engagement – Childcare | .06 | .06 | -.05 |
| Possible task engagement – Work | .15^†^ | -.05 | -.12 |
| Possible task engagement – Routine household tasks | -.05 | .12 | .29* |
| Possible task engagement – Non-routine household tasks | -.14^†^ | -.08 | -.05 |
| Adjusted *R*^2^ | .04 | .06 | .21 |

*Notes.* *** *p* < .001, ** *p* < .01, * *p* < .05, † *p* < .10.
